# Immunogenicity profiling and distinct immune response in liver transplant recipients vaccinated with SARS-CoV-2 inactivated vaccines

**DOI:** 10.3389/fimmu.2022.954177

**Published:** 2022-09-14

**Authors:** Binwei Duan, Gongming Zhang, Wenjing Wang, Jiming Yin, Mengcheng Liu, Jing Zhang, Dexi Chen, Yabo Ouyang, Guangming Li

**Affiliations:** ^1^ Department of General Surgery Center, Beijing YouAn Hospital, Capital Medical University, Beijing Institute of Hepatology, Beijing, China; ^2^ Clinical Center for Liver Cancer, Capital Medical University, Beijing, China; ^3^ Beijing Precision Medicine and Transformation Engineering Technology Research Center of Hepatitis and Liver Cancer, Beijing, China

**Keywords:** SARS-CoV-2 inactivated vaccines, liver transplant recipient, neutralizing antibodies, CD3+ CD19+ cell, CXCL10

## Abstract

SARS-CoV-2 vaccination has been recommended for liver transplant (LT) recipients. However, our understanding of inactivated vaccine stimulation of the immune system in regulating humoral and cellular immunity among LT recipients is inadequate. Forty-six LT recipients who received two-dose inactivated vaccines according to the national vaccination schedule were enrolled. The clinical characteristics, antibody responses, single-cell peripheral immune profiling, and plasma cytokine/chemokine/growth factor levels were recorded. Sixteen (34.78%) LT recipients with positive neutralizing antibody (nAb) were present in the Type 1 group. Fourteen and 16 LT recipients with undetected nAb were present in the Type 2 and Type 3 groups, respectively. Time from transplant and lymphocyte count were different among the three groups. The levels of anti-RBD and anti-S1S2 decreased with decreasing neutralizing inhibition rates. Compared to the Type 2 and Type 3 groups, the Type 1 group had an enhanced innate immune response. The proportions of B, DNT, and CD3+CD19+ cells were increased in the Type 1 group, whereas monocytes and CD4+ T cells were decreased. High CD19, high CD8+CD45RA+ cells, and low effector memory CD4+/naïve CD4+ cells of the T-cell populations were present in the Type 1 group. The Type 1 group had higher concentrations of plasma CXCL10, MIP-1 beta, and TNF-alpha. No severe adverse events were reported in all LT recipients. We identified the immune responses induced by inactivated vaccines among LT recipients and provided insights into the identification of immunotypes associated with the responders.

## Introduction

Solid organ transplant (SOT) recipients are at a high risk of SARS-CoV-2 infection and its severe outcomes (1, 2). Liver transplant (LT) recipients or other immunocompromised patients are a highly vulnerable patient population, requiring SARS-CoV-2 vaccination, as recommended by some societies (3, 4). Due to immunosuppressive treatment effects, lower immune response and fewer detectable SARS-CoV-2 antibodies to the SARS-CoV-2 mRNA vaccine among SOT recipients than among the immunocompetent population have been documented (5–12). Some studies have reported lower immunological and poor antibody response to mRNA-based vaccines among LT recipients (11, 13). Inactivated vaccines have proven to be strongly immunogenic and highly efficient in preventing severe coronavirus disease (COVID-19) in immunocompetent individuals (14–16). However, knowledge of inactivated vaccine-induced humoral and cellular responses in SOT recipients, especially LT recipients, remains poorly understood.

Vaccines may prevent infection and its unfavorable effects by inducing robust virus neutralizing antibody (nAb) responses, which are crucial for shaping both humoral and cellular protective immunity during the early response to vaccination (17, 18). In addition to nAb, T cells are critically necessary for clearing viral infections and effective vaccination to maintain extensive and lasting antiviral immunity (19, 20). Our previous study confirmed that T-cell immune response changed during disease progression in patients with COVID-19 (21). Cytokines and chemokines play a key role in the development and maintenance of immunity in response to infection and vaccination. Early cytokine and chemokine signatures may be used to monitor effective vaccination; they have been proposed as guides for optimizing the efficacy of mRNA vaccination strategies (22).

Knowledge of the two-dose inactivated SARS-CoV-2 vaccine-induced immune response in LT recipients remains poor, especially the comprehensive difference in humoral and cellular responses between responders and non-responders. Defining the nature of immune response after SARS-CoV-2 vaccination could help identify biomarkers for predicting the effective application of vaccines in LT recipients. In this study, we used the systems vaccinology approach to comprehensively profile the innate and adaptive immune responses of LT recipients who were vaccinated with the two-dose inactivated SARS-CoV-2 vaccine. Additionally, we evaluated the clinical characteristics, antibody responses, single-cell peripheral immune profiling, and plasma cytokine/chemokine/growth factor levels among LT recipients with SARS-CoV-2 inactivated vaccination.

## Patients and methods

### Patient population and study design

**Figure 1:**
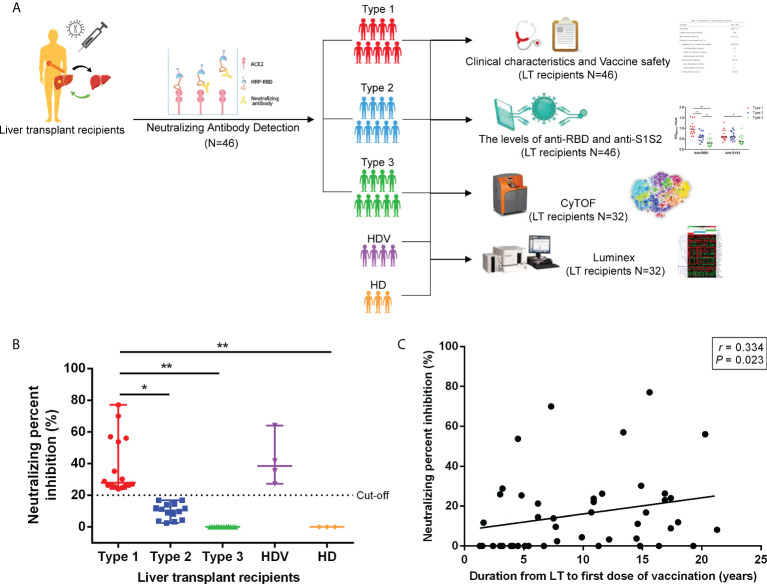
Schematic workflow and SARS-CoV-2 neutralizing antibody detection after vaccination. **(A)** Schematic description of LT recipient groups and blood sample experiments. **(B)** Neutralizing antibody detection in plasma of LT recipients after two-dose inactivated vaccination. Cutoff value equal to 20% signal inhibition. Neutralizing percent inhibition (NPI) of sample ≥20% indicated the presence of Anti-SARS-CoV-2 neutralizing antibodies, whereas NPI < 20% indicated the absence of neutralizing antibodies. **(C)** Correlation between the duration from LT to first dose of vaccination and NPI. *p*-Values (two-sided) and r values are based on Spearman’s rank test. LT, liver transplant. *p < 0.05, **p < 0.01.

This study was an observational study conducted among LT recipients who had received two scheduled doses of the inactivated vaccines (CoronaVac or BBIBP-CorV) within 8 weeks, according to the national vaccination protocol. The participants were recruited from an online survey. Three healthy donors without vaccination (HD) and four healthy donors vaccinated with the inactivated vaccine (HDV) were recruited as the no vaccination healthy controls and vaccination healthy controls, respectively. Blood samples from LT recipients and HDVs were obtained within 4–8 weeks after administration of the second dose of the vaccine for CyTOF and cytokine detection. The exclusion criteria included age <18 years and history of COVID-19 diagnosis. All the clinical data of LT recipients within 4 weeks before the first dose of the vaccine were retrospectively reviewed. **Figure 1A** shows the study flow diagram for the study. This study was approved by the Ethics Committee of Beijing YouAn Hospital ([2021]083), and all participants provided written informed consent.

### Anti-SARS-CoV-2 neutralizing antibody detection

Anti-SARS-CoV-2 neutralizing antibody levels were determined by competitive enzyme-linked immunosorbent assay (ELISA) using the Anti-SARS-CoV-2 Neutralizing Antibody Titer Serologic Assay Kit (ACROBiosystems, Newark, DE, USA), according to the manufacturer’s instructions. Briefly, the microplate in the kit was pre-coated with human ACE2 protein. Plasma samples, positive control, and negative control were added to the wells, followed by the addition of HRP-SARS-CoV-2 Spike RBD. After incubation, the wells were washed, and the substrate solution was added to the wells. The reaction was terminated by the addition of a stop solution, and the intensity of absorbance was measured at 450 nm/630 nm. The neutralizing antibodies in the samples competed with ACE2 for HRP-SARS-CoV-2 Spike RBD binding. The intensity of the assay signal decreased proportionally with the concentration of Anti-SARS-CoV-2 neutralizing antibodies. The cutoff value was set at 20% of signal inhibition. A neutralizing percent inhibition (NPI) of sample ≥20% indicated that Anti-SARS-CoV-2 neutralizing antibodies were present, whereas NPI <20% indicated the absence of neutralizing antibodies.

### Detection of antibody titer against SARS-CoV-2

The titers of antibodies against structural proteins, RBD and S1S2, were determined using the indirect ELISA kit (Sino Biological, Beijing, China) according to the manufacturer’s instructions, as previously described (23). Each value obtained was an average of three independent biological replicates.

### Cytokine/chemokine/growth factor detection with Luminex kits

Plasma cytokine/chemokine/growth factor concentrations were measured by the Luminex bead-based MILLIPLEX assay using MILLIPLEX^®^ Human Cytokine/Chemokine/Growth Factor Panel A (Millipore, Billerica, MA, USA) with a FlexMAP3D (Luminex) platform. Cytokine production data were analyzed using the xPONENT software, following the manufacturer’s instructions (24). The panel simultaneously analyzed 48 multiple cytokine, chemokine, and growth factor biomarkers, including sCD40L, EGF, eotaxin, FGF-2, Flt-3, ligand, fractalkine, G-CSF, GM-CSF, GRO-alpha, IFN-alpha2, IFN-gamma, IL-1 alpha, IL-1beta, IL-1RA, IL-2, IL-3, IL-4, IL-5, IL-6, IL-7, IL-8, IL-9, IL-10, IL-12 (p40), IL-12 (p70), IL-13, IL-15, IL-17A, IL-17E/IL-25, IL-17F, IL-18, IL-22, IL-27, IP-10, MCP-1, MCP-3, M-CSF, MDC, MIG, MIP-1 alpha, MIP-1 beta PDGF-AA, PDGF-AB/BB, RANTES, TGF-alpha, TNF-alpha, TNF-beta, and VEGF-A.

### Mass cytometry

Peripheral blood mononuclear cells (PBMCs) of participants were incubated with 1 μM of cisplatin (198-Pt, Fluidigm, South San Francisco, CA, USA) for 2 min for viability evaluation by mass cytometry. Cells were then fixed for 15 min at room temperature with Fix I (Fluidigm) buffer and washed three times with Cell Staining Buffer (CSB) for further analysis. A palladium isotope barcoding kit was applied to minimize inter-sample staining variation. Briefly, each sample was counted and diluted to 1 × 10^6^ cells/ml before being labeled with a unique combination of three palladium isotopes. Thereafter, 20 samples from different groups were mixed. The purified antibodies, as shown in **Supplementary Table 1**, were conjugated with the Multi-Metal MaxPar Kit (Fluidigm). All metal-conjugated antibodies were titrated for optimal concentration before use. The mixed cells were stained with surface markers, such as CD3, CD4, and CD8, for 30 min. They were then permeabilized with ice-cold methanol (80%) for 15 min. After three washes with CSB, cells were incubated with the remaining antibodies. After three washes with CSB followed by staining with Intercalator-Ir (Fluidigm) at 4°C overnight, the samples were washed three times with ultrapure water. Thereafter, the cells were resuspended in ultrapure water containing 10% of EQ Four Element Calibration Beads (Fluidigm). Lastly, the data were obtained from the Helios mass cytometer (Fluidigm).

### Mass cytometry data analysis

All.fcs files were uploaded into Cytobank. Data cleaning was performed, as described previously. The population of single living cells was exported as.fcs files for further analysis (25). Files were loaded into R (http://www.rstudio.com), and the arcsinh transform was performed to signal intensities of all channels. PhenoGraph analysis was performed, as previously described (26).

### Statistical analysis

All statistical analyses were performed using the SPSS software package (version 23.0; SPSS Inc., USA). Comparisons of differences among groups were performed using the Kruskal–Wallis test followed by multiple comparisons with pairwise, chi-square, or Fisher’s exact test. Statistical significance was set at *p* < 0.05 for two-sided tests. Correlation analyses were performed using Spearman’s rank test.

## Results

### Patient characteristics

Forty-six LT recipients were recruited from 11 hospitals. They had received inactivated vaccines (CoronaVac or BBIBP-CorV) according to the national vaccination schedule. The baseline characteristics of the cohort are shown in **Table 1**. The most common etiology of liver disease was hepatitis B virus-related liver disease (36, 78.3%), followed by alcoholic liver disease (four, 8.7%), primary biliary cirrhosis (three, 6.5%), and Wilson’s disease (three, 6.5%). A proportion of 58.7% (27/46) of patients received two kinds of immunosuppressants, and 39.1% (18/46) of them received a single regimen. One participant received the triple-drug regimen, which comprised a calcineurin inhibitor, glucocorticoid, and antimetabolite. The median time from LT to the first vaccination was 7.7 years (range, 1.3–21.3 years).

**Table 1:** Characteristics of LT recipients.

Variables	All (n = 46)
Age (years)	58 (28–71)
Gender (male versus female)	42:4
BMI, median (mean ± SD)	23.6 ± 2.5
Etiology of liver disease pre-LTx	
Hepatitis B virus-related liver disease	36 (78.3%)
Decompensated cirrhosis	18
Acute-on-chronic liver failure	5
Hepatocellular carcinoma	13
Alcoholic liver diseases	4 (8.7%)
Decompensated cirrhosis	3
Hepatocellular carcinoma	1
Primary biliary cirrhosis	3 (6.5%)
Wilson’s disease	3 (6.5%)
Complicated with HCV	1
Immunosuppression	
Calcineurin inhibitors	38 (82.6%)
Tacrolimus	36
Cyclosporine	2
Sirolimus	12 (26.1%)
MMF/MPA	25 (54.3%)
Prednisone	2 (4.3%)
Comorbidities	
Diabetes	12 (26.1%)
Hypertension	15 (32.6%)
Chronic myelocytic leukemia	1
HGB (g/L)	148.0 ± 11.6
White blood cell count (×10^9^/L)	5.99 (3.48–11.09)
Neutrophil count (×10^9^/L)	3.44 (2.09–9.14)
Lymphocyte count (×10^9^/L)	1.69 (0.64–4.51)
Platelet count (×10^9^/L)	177 (109–341)
eGFR, ml/min/1.73 m^2^	93.33 ± 15.15
Duration from LT to first dose of vaccination (years)	7.7 (1.3–21.3)
Interval between two doses (days)	24 (15–46)
Type of inactivated vaccine (CoronaVac: BBIBP-CorV)	33:13

LT, liver transplant; BMI, body mass index; HCV, hepatitis C virus; MMF, mycophenolate mofetil; MPA, mycophenolic acid; HGB, hemoglobin; eGFR, estimated glomerular filtration rate.

### Difference in neutralizing inhibition rate among liver transplant recipients

To examine the ability of plasma antibodies to interfere with ACE2–RBD interaction, a competitive SARS-CoV-2 serology assay was performed. In this assay, plasma antibodies were added to ELISA plates precoated with the SARS-CoV-2 RBD protein, followed by the addition of the human ACE2 protein. A specific neutralizing antibody against SARS-CoV-2 RBD was used as a reference. After the second dose, 16 (34.78%) participants had a positive neutralizing antibody reaction (**Figure 1B**). No neutralizing antibodies were detected in 30 (65.22%) patients. In those with positive neutralization post-second dose, the median level of percent inhibition was 26.25% (21.25%–77.02%). Thereafter, these LT recipients were divided into three groups according to the NPI findings. They included the detectable Anti-SARS-CoV-2 neutralizing antibody group (Type 1, 16 patients, NPI ≥ 20%) and undetectable neutralizing antibody groups (Type 2, 14 patients, 20% > NPI > 0%; Type 3, 16 patients, NPI ≤ 0%). As shown in **Figure 1B**, the NPI of Type 1 was higher than that of Type 2 (*p* = 0.032), Type 3 (*p* < 0.001), and HD (*p* = 0.006). The following analyses were performed based on these groups. The clinical and laboratory data of LT recipients with detectable neutralizing antibodies and NPI were compared (**Table 2**). Patients with detectable neutralizing antibodies in the negative group were more frequently treated with mycophenolate mofetil/mycophenolic acid (MMF/MPA) than those in the positive group (20/10 vs 5/11, *p* = 0.022). Duration from LT to first dose of vaccination (*p* = 0.013) and lymphocyte count (*p* = 0.015) showed a significant difference among the Type 1, Type 2, and Type 3 groups, which indicated longer post-transplant time (*p* = 0.015) and higher lymphocyte count (*p* = 0.014) in Type 1 than Type 3. Duration from LT to first dose of vaccination was correlated with NPI (**Figure 1C**; Spearman’s r = 0.334, *p* = 0.023), while no significant correlation between lymphocyte count and NPI was found (Spearman’s r = 0.281, *p* = 0.068). No significant difference in sex, body mass index, etiology of liver disease, source of vaccine, estimated glomerular filtration rate (eGFR), and comorbidities were noted in neutralizing antibody response.

**Table 2:** **|** Comparison of recipients in neutralizing antibody and neutralizing percent inhibition.

Variables	Neutralizing antibody		*p*-Value	Neutralizing percent inhibition			p-Value
	Negative(n = 30)	Positive(n = 16)			Type 1(n = 16)	Type 2(n = 14)		Type 3(n = 16)	
Age (years)	56.75 ± 9.25	55.38 ± 10.25	0.648	55.3 ± 10.2	58.0 ± 9.3	55.7 ± 9.4	0.731
Gender	27:3	15:1	0.667	15:1	13:1	14:2	0.797
BMI	23.84 ± 2.55	23.27 ± 2.41	0.467	23.27 ± 2.41	24.46 ± 2.43	23.30 ± 2.60	0.343
Duration from LT to first dose of vaccination (years)	7.7 (1.3–21.3)	10.9 (3.0–20.3)	0.406	10.9 (3–20.3)^#^	13.4 (3.6–21.3)	4.3 (1.3–19.3)^#^	0.013^*^
Interval between two doses (days)	23 (15–46)	25 (21–33)	0.065	25 (21–33)	23 (21–46)	24 (15–39)	0.175
CoronaVac/BBIBP-CorV	20/10	13/3	0.295	13/3	7/7	13/3	0.096
Calcineurin inhibitors or not	24/6	12/4	0.695	12/4	11/3	13/3	0.912
Tacrolimus daily dose (mg/day)	1.0 (0.5–6.0)	1.0 (0.1–2.0)	0.363	1.0 (0.1–2.0)	1.0 (0.5–2.5)	1.0 (0.5–6.0)	0.583
MMF/MPA or not	20/10	5/11	0.022^*^	5/11	9/5	11/5	0.069
HGB (g/L)	147.6 ± 10.7	148.9 ± 13.7	0.889	148.0 ± 13.5	147.0 ± 8.7	148.0 ± 12.0	0.965
White blood cell count (×10^9^/L)	6.06 (3.48–11.09)	5.93 (3.70–9.70)	0.872	5.98 (3.70–9.70)	6.33 (3.48–10.66)	5.68 (3.75–11.09)	0.546
Neutrophil count (×10^9^/L)	3.62 (2.48–9.14)	3.23 (2.09–7.03)	0.878	3.17 (2.09–7.03)	3.49 (2.39–6.05)	3.76 (2.28–9.14)	0.986
Lymphocyte count (×10^9^/L)	1.45 (0.64–4.51)	1.92 (0.79–3.62)	0.516	2.02 (0.79–3.62)^#^	2.27 (0.64–4.09)	1.23 (0.96–4.51)^#^	0.015^*^
Platelet count (×10^9^/L)	177 (115–288)	177 (109–341)	0.310	177 (109–341)	175 (115–288)	181 (119–248)	0.589
eGFR, ml/min/1.73 m^2^	91.04 ± 14.89	97.93 ± 15.14	0.089	99.13 ± 15.30	96.20 ± 14.77	87.17 ± 14.21	0.120
Diabetes or not	10/20	2/14	0.125	1/5	5/9	5/11	0.696

BMI, body mass index; LT, liver transplant; MMF, mycophenolate mofetil; MPA, mycophenolic acid; HGB, hemoglobin; eGFR, estimated glomerular filtration rate.

^*^p < 0.05.

^#^There are significant differences between the two groups using Kruskal–Wallis test followed by multiple comparisons with pairwise.

### Enhanced response of antibodies against SARS-CoV-2 structural proteins in patients with higher neutralizing inhibition rate

**Figure 2:**
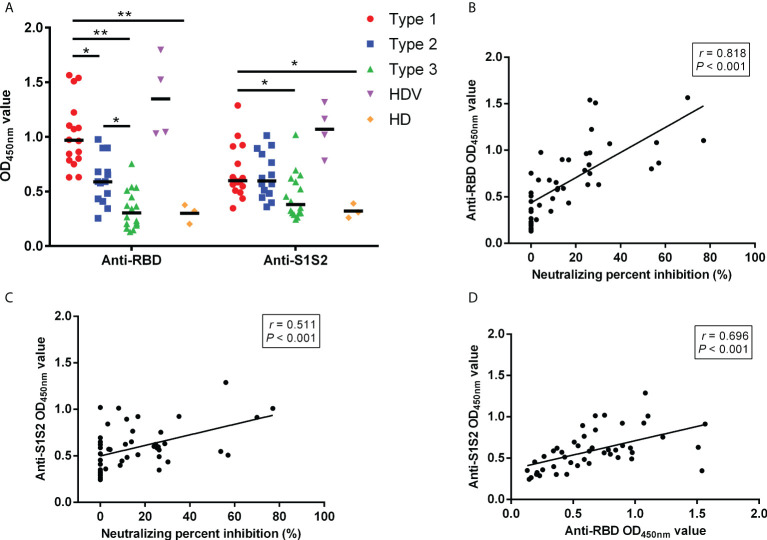
Comparison of antibody responses between different groups. **(A)** Comparison of anti-RBD and anti-S1S2 antibody responses among the Type 1, Type 2, and Type 3 groups. Correlation between anti-RBD and anti-S1S2 levels. **(B)** NPI and anti-RBD levels. **(C)** NPI and anti-S1S2 levels. **(D)** *p*-Values (two-sided) and r values are based on Spearman’s rank test. NPI, neutralizing percent inhibition. *p < 0.05, **p < 0.01.

Furthermore, we investigated the response of antibodies against RBD and S1S2 among the Type 1, Type 2, and Type 3 groups. The anti-RBD and anti-S1S2 levels gradually decreased with decreasing neutralizing inhibition rates among groups. The anti-RBD antibody levels were higher in the Type 1 group than in the Type 2 group (*p* = 0.018), Type 3 group (*p* < 0.001), and HD (*p* = 0.002) (**Figure 2A**). Additionally, the anti-RBD level was higher in the Type 2 group than in the Type 3 group (*p* = 0.042). Higher anti-S1S2 titers were detected in the Type 1 group than in the Type 3 group (*p* = 0.030) and HD (*p* = 0.015), although no differences were present between Type 1 and Type 2 (*P* = 1.000) or Type 2 and Type 3 (*p* = 0.059). The potential correlation between anti-RBD/anti-S1S2 levels and NPI was evaluated. NPI had a high correlation with anti-RBD level (**Figure 2B**; Spearman’s r = 0.818, *p* < 0.001). Additionally, NPI was correlated with anti-S1S2 level (**Figure 2C**; Spearman’s r = 0.511, *p* < 0.001), and the anti-RBD level was positively correlated with anti-S1S2 level (**Figure 2D**; Spearman’s r = 0.696, *p* < 0.001).

### Induction of innate immune responses

**Figure 3:**
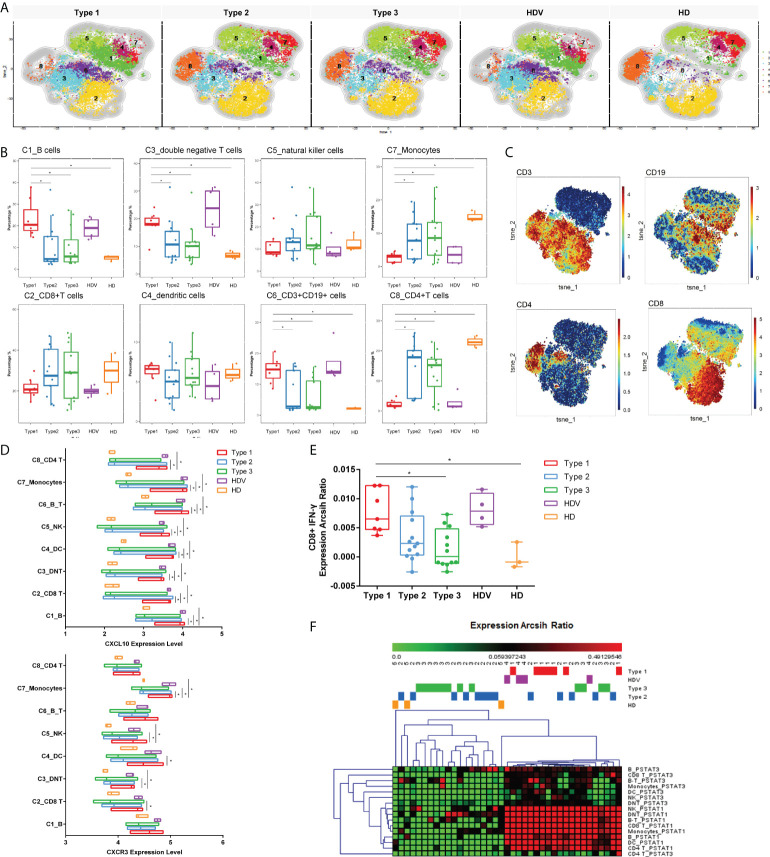
Innate immune responses induced by inactivated vaccination among LT recipients. **(A)** CyTOF-identified cell clusters from PBMCs visualized by t-distributed stochastic neighbor embedding (t-SNE). **(B)** Percentage of each cluster among five groups. Boxes represent interquartile ranges (IQRs). Each dot represents an individual group: Type 1 (red), Type 2 (blue), Type 3 (green), HDV (purple), or HD (orange). **(C)** viSNE projections of expression of the indicated proteins. **(D)** The expression level of CXCL10 and CXCR3 in eight cell clusters. Line at median of groups. **(E)** IFN-γ expression levels measured and compared among five groups in CD8+ cells. **(F)** Heatmap of expression arcsih ratio of pSTAT3 and pSTAT1 levels. Significance was determined using Kruskal–Wallis test, followed by multiple pairwise comparisons. Statistical significance was set at a two-sided *p*-value <0.05 and adjusted *p* < 0.05. *p < 0.05. LT, liver transplant; PBMCs, peripheral blood mononuclear cells; HDV, healthy donors vaccinated with the inactivated vaccine; HD, healthy donors without vaccination.

To reveal differences in cell-type compositions after vaccination among groups, we calculated the relative percentages of cell types in PBMCs of 32 LT recipients (Type 1, 7 patients; Type 2, 13 patients; Type 3, 12 patients) using CyTOF data. After surface markers were combined by unsupervised clustering, CD45+ PBMCs were divided into eight major cell types (**Figures 3A–C**), including C1_B cells (CD19+), C2_CD8+T cells (CD8+ CD3+), C4_dendritic cells (DCs, HLA-DR+), C5_natural killer cells (NK, CD56+), C7_Monocytes (CD14+HLA-DR+CD16−), C8_CD4+T cells (CD4+ CD3+), C6_CD3+CD19+ (B-T) cells, and C3_double negative T cells (DNT). The frequencies of B, DNT, and CD3+CD19+ cells were higher in the Type 1 group than in the Type 2, Type 3, and HD groups. However, they were decreased in monocytes and CD4^+^ T cells in the Type 1 group (**Figure 3B**). The frequencies of these eight cell types were not different between the Type 1 group and HDVs. Additionally, CXCR3 was highly expressed in CD8 T, DNT, DC, NK, and monocytes in the Type 1 group, and the expression level of its ligand, CXCL10, was elevated among all eight clusters in the Type 1 group (**Figure 3D**). IFN-γ expression level was higher in CD8 T cells of the Type 1 group than in those of the Type 3 or HD group (**Figure 3E**). Compared to the Type 2, Type 3, and HD groups, the Type 1 group had enhanced levels of phosphorylated (p)STAT1 and pSTAT3 in multiple cell types (**Figure 3F**). These data suggest that a heightened innate immune response was induced after secondary immunization in LT recipients in the Type 1 group, compared to those in the Type 2 and Type 3 groups.

### Identifying circulating T-cell responses with vaccine effectiveness in liver transplant recipients

**Figure 4:**
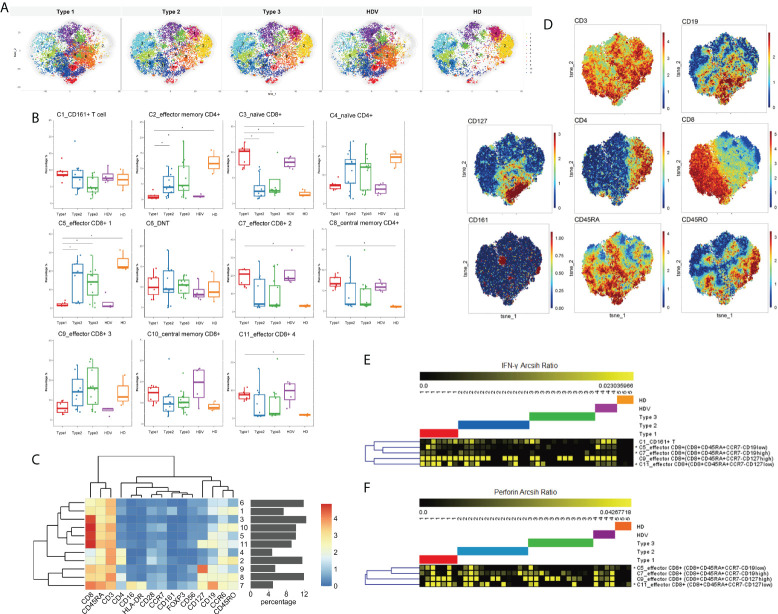
T-cell activation in subsets of LT recipients is associated with vaccination responder. **(A)** CyTOF-identified cell clusters from CD3+ cells visualized by t-SNE. **(B)** Percentage of each cluster among five groups. Boxes represent interquartile ranges (IQRs). Each dot represents an individual groups: Type 1 (red), Type 2 (blue), Type 3 (green), HDV (purple), or HD (orange). **(C)** Heatmap showing expression patterns of various markers, stratified by FlowSOM clusters. Heat scale calculated as column z-score of mean fluorescence intensity (MFI). **(D)** viSNE projections of expression of the indicated proteins. IFN-γ **(E)** and perforin **(F)** expression levels measured and compared in four kinds of effector CD8+ cells among five groups. Significance was determined using Kruskal–Wallis test, followed by multiple pairwise comparisons. Statistical significance was set at a two-sided *p*-value <0.05 and adjusted *p* < 0.05. *p < 0.05. Percentages represent proportion of each identified cluster of all analyzed cells. LT, liver transplant; t-SNE, t-distributed stochastic neighbor embedding; HDV, healthy donors vaccinated with the inactivated vaccine; HD, healthy donors without vaccination.

Thereafter, we investigated the immune features of T lymphocytes (CD3+ cells), with respect to activation and differentiation, and identified the CD3+ CD19+ subsets of circulating T cells after two doses of the inactivated vaccine in LT recipients. Eleven T-cell populations were clustered (**Figures 4A–D**); they included CD161+ T cell (C1, CD3+ CD161+), effector memory CD4+ (C2, CD4+CD45RO+CCR7−), naïve CD8+ (C3, CD8+CD45RA+CCR7+), naïve CD4+ (C4, CD4+CD45RA+CCR7+), effector CD8+ 1 (C5, CD8+CD45RA+CCR7−CD19^low^), DNT (C6), effector CD8+ 2 (C7, CD8+CD45RA+CCR7−CD19^high^), central memory CD4+ (C8, CD4+CD45RO+CCR7+), effector CD8+ 3 (C9, CD8+CD45RA+CCR7−CD127^high^), central memory CD8+ (C10, CD8+CD45RO+CCR7+), and effector CD8+ 4 (C11, CD8+CD45RA+CCR7−CD127^low^) cells. Among the T-cell populations, the CD19^high^ CD8+CD45RA+ cell population increased, whereas the effector memory CD4+ and naïve CD4+ cells decreased in the Type 1 group, compared to the Type 2, Type 3, and HD groups (**Figure 4B**). Cluster 7, CD19^high^ CD8+CD45RA+ cells, increased and was characterized by high CD45RA, CD8, and CD19 expression; moderate CD16, HLA-DR, and CD127 expression; and low CD4 expression (**Figure 4C**). Furthermore, the frequencies of C8_central memory CD4+, C9_effector CD8+, and C11_effector CD8+ cells were higher in the Type 1 group than in the HD group (**Figure 4B**). Notably, the three vaccination-specific cell subsets had co-expression of CD19 and CD127. Although the frequency of naïve CD8+ cells was not different among the five groups, it was decreased in the Type 1 and HDV groups (**Figure 4B**). Thereafter, IFN-γ and perforin were expressed among the four kinds of effector CD8+ in the different groups. IFN-γ expression levels in C5_effector CD8+, C7_effector CD8+, and C11_effector CD8+ cells were higher in the Type 1 group than in the Type 3 group. However, no difference in C9_effector CD8+ cells was present between the Type 1 and Type 2/Type 3 groups (**Figure 4E**). The Type 1 group had an increased perforin expression level than the Type 3 group in only the C5_effector CD8+ and C11_effector CD8+ subsets (**Figure 4F**).

### Cytokine/chemokine/growth factor profile induced by the two-dose inactivated vaccine in liver transplant recipients

**Figure 5:**
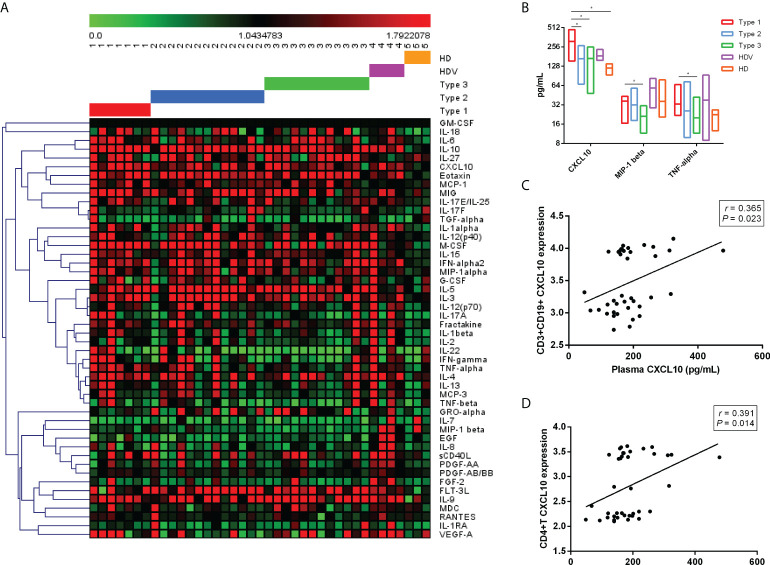
Plasma cytokine/chemokine/growth factor levels after two-dose vaccination in LT recipients. **(A)** Heatmaps representing the 48 cytokine/chemokine/growth factor levels among the Type 1, Type 2, Type 3, HDV, and HD groups. **(B)** Higher concentrations of plasma CXCL10, MIP-1 beta, and TNF-alpha in Type 1. Correlation between plasma CXCL10 level and CD3+ CD19+ CXCL10 expression **(C)** and CD4+ CXCL10 levels **(D)**. *p*-Values (two-sided) and r values are based on Spearman’s rank test. LT, liver transplant; HDV, healthy donors vaccinated with the inactivated vaccine; HD, healthy donors without vaccination. *p < 0.05.

To further investigate the cytokine signature induced by the two-dose inactivated vaccine, we measured plasma cytokines in 32 vaccinated individuals. Of the 48 cytokines detected, CXCL10 (*p* = 0.021), MIP-1 beta (*p* = 0.009), and TNF-alpha (*p* = 0.034) were significantly different among the Type 1, Type 2, and Type 3 groups (**Figure 5A**). Although CXCL10 level was higher in the Type 1 group than in the Type 2 group (*p* = 0.040), Type 3 group (*p* = 0.031), and HD group (*p* = 0.009), MIP-1 beta (*p* = 0.013) and TNF-alpha (*p* = 0.032) levels were only higher in the Type 1 group than in the Type 3 group (**Figure 5B**). Additionally, CXCL10 levels were higher in the Type 1 group than in the HD group (**Figure 5B**). The potential correlations between CXCL10 levels of all cases and the corresponding expression levels of eight immune cells were analyzed using Spearman’s rank order correlation test (**Figures 5C, D**). We found that CXCL10 level was positively correlated with CXCL10 expression in CD3+ CD19+ cells (Spearman’s r = 0.365, *p* = 0.023) and CD4+ T cells (Spearman’s r = 0.391, *p* = 0.014). No significant correlation was observed between CXCL10 levels and CXCL10 expression in the rest of the B cells (Spearman’s r = 0.310, *p* = 0.055), CD8+ T cells (Spearman’s r = 0.307, *p* = 0.057), DCs (Spearman’s r = 0.202, *p* = 0.218), NK (Spearman’s r = 0.267, *p* = 0.101), monocytes (Spearman’s r = 0.272, *p* = 0.094), and DNT cells (Spearman’s r = 0.288, *p* = 0.076).

### Vaccine safety

Safety analysis was completed for all participants who completed the vaccine diary after receiving both vaccine doses (**Supplementary Table 2**). Overall, the vaccines were well-tolerated. Local events with associated pain and swelling at the injection site were most commonly reported. Systemic events included fatigue, myalgia, and headache. No episodes of organ rejection were recorded 6 weeks after the second vaccine dose.

## Discussion

Due to the life-long immunosuppression and high prevalence of comorbidities, LT recipients are at a high risk of SARS-CoV-2 infection (1, 3). Few studies have evaluated LT recipients for antibody responses to mRNA vaccines (11, 13). The mechanism of cellular response after vaccination in LT recipients remains unclear. In this study, LT recipients who had been vaccinated with inactivated vaccines (CoronaVac or BBIBP-CorV) were divided into three groups according to the nAb detection results. Thereafter, we comprehensively compared the innate and adaptive immune responses among the different groups. Our study provides important insight into how LT recipients respond to the COVID-19 vaccine. ().

In our study, as expected, LT recipients had lower immunogenicity toward SARS-CoV-2 inactivated vaccines, similar to the reported response to mRNA vaccines (11). Risk factors for negative serology among solid organ transplant recipients include MMF treatment (as described in this study), high-dose steroid use, triple therapy immunosuppression, old age, and reduced eGFR (10, 11, 27). The predominantly used immunosuppressive anti-metabolite, MMF/MPA, impairs both seroconversion rate and IgG and RBD-IgG titers in organ transplant recipients 2 months after SARS-CoV-2 mRNA vaccination (10, 12, 28). A recent meta-analysis showed that MPA/MMF weakened antibody response to the SARS-CoV-2 vaccine in adult solid organ transplant recipients (29). MMF/MPA may delay humoral response with significant antibody decline in kidney transplant recipients after SARS-CoV-2 mRNA vaccination (30). However, the NPI-related factors in this study included duration between the time of LT and the first dose of vaccination and lymphocyte count prior to vaccination. Overall, the vaccines were well-tolerated, and no major adverse events or graft rejections were recorded after vaccination.

LT recipients mount a poor antibody response to mRNA SARS-CoV-2 vaccines. IgG antibody titer and neutralizing antibodies were present in 61% and 47.5% of LT recipients, respectively, as reported in two studies (11, 13). In this study, 34.78% of LT recipients developed positive neutralizing antibodies after receiving the two-dose inactivated vaccines, which was consistent with findings from previous reports. Among the three types of LT recipients grouped by nAb results, the Type 1 (nAb+) group had higher anti-RBD and anti-S1S2 titers than the Type 2 (nAb−) and Type 3 (nAb−) groups. According to previous reports, a strong correlation exists between levels of RBD-binding antibodies and SARS-CoV-2 nAbs in patients with SARS-CoV-2 (31, 32). Similarly, this study confirmed a strong correlation between NPI and anti-RBD/anti-S1S2 levels in LT recipients. These results suggest that proportional LT recipients could promote robust humoral responses through two-dose inactivated vaccines.

One of the major concerns in transplant patients is the difference in cellular immunity between responders and non-responders. Therefore, the immune response characteristics related to vaccine efficacy in LT recipients should be elucidated. We investigated the peripheral single-cell immune spectrum after vaccination in LT recipients using high-parameter CyTOF analysis to evaluate the phenotypes of their peripheral immune cells. Notably, similar immune cell frequencies were observed in the Type 1 and HDV groups. The proportions of B cells, DNT cells, and CD3+CD19+ cells were higher in the Type 1 group than in the Type 2, Type 3, and HD groups. Circulating B cells increase in numbers after vaccination against SARS-CoV-2, implying that B cells rapidly proliferate and expand in a good immune response (33). Additionally, the percentages of DNT cells were found to be significantly increased in patients with COVID-19 (34). Although increased to a certain degree in Type 2 and Type 3 groups, interestingly, CD3+CD19+ cells characterized by high CD45, CD3, and CD19 expression levels were mainly found in vaccinated individuals in the Type 1 and HDV groups relative to the HD group. The CD3+CD19+ cells were present in peripheral blood samples from patients with HIV/mycobacterium tuberculosis infection and patients with cancer, reflecting the adaptive immune landscape (35, 36). Combining findings from our study and other studies, although all the studies showed that CD3+CD19+ cells were present commonly in the peripheral blood of healthy donors, the percentage of CD3+CD19+ cells in patients with cancer/vaccination population was significantly higher than that of CD3+CD19+ cells in healthy donors. It is a special subset of immune cells that probably plays a complex role in an intermediate state. We noticed that monocyte and CD4+ T-cell proportions were significantly decreased in the Type 1 and HD groups. CD4+ T-cell response was quicker than CD8+ T-cell response after two doses of the vaccine. CD8 T cells mostly produced IFN-γ, which were detected in CD8 T cells of the Type 1 group, compared to the Type 3 and HD groups. IFN-γ activates the Janus kinase (JAK)-signal transducer and activator of transcription (STAT) signaling pathway, resulting in the upregulation of STAT1 transcriptional targets (37). In this study, pSTAT1 and pSTAT3 expression levels were higher in the Type 1 group than in the Type 2, Type 3, and HD groups. These results suggest that LT recipients in the Type 1 group had a heightened innate immune response after secondary immunization, compared to LT recipients in the Type 2 and Type 3 groups.

More specifically, we used high-dimensional flow cytometry to perform immune profiling of T-cell populations. Several key findings included the high CD19, high CD8+CD45RA+ cell, and low effector memory CD4+ and naïve CD4+ cells in the Type 1 group. Additionally, central memory CD4+ cells and a fraction of effector CD8+ cells were specifically increased in vaccinated populations, with co-expression of CD19 and CD127. CD127 expression, as a feature of memory in T cells, promotes the survival and maintenance of long-lived memory T cells; CD127 is expressed on effector CD8+ T cells (38, 39). The elevated IFN-γ and cytotoxic molecules perforin expression level of effector CD8+ T cells in the Type 1 group, determined in our study, indicated the immune response induced by vaccination among LT recipients.

Cytokine modulation could be a marker of successful vaccination resulting in efficient antibody development (22). Our analysis of the peripheral levels of cytokines, chemokines, and inflammation markers suggested that CXCL10, MIP-1 beta, and TNF-alpha were associated with the effective immune response to the two-dose inactivated SARS-CoV-2 vaccine in LT recipients. Surprisingly, the expression level of CXCL10 was elevated among all eight immune cell clusters in the Type 1 group. Among them, the CXCL10 level was positively correlated with CXCL10 expression in CD19+CD3+ cells and CD4+ T cells. Correspondingly, plasma CXCL10 levels were used to monitor effective vaccination and guide the efficacy of mRNA vaccination strategies (22). The chemokine, CXCL10, is often released in the context of inflammation by many immune cells and promotes the chemotaxis of CXCR3+ cells, which are mainly activated T and B lymphocytes (40, 41). As previously reported, TNF-alpha was induced after the second mRNA vaccination, which could play a role with CXCL10 in the rapid recruitment and stimulation of effector immune cells (22). MIP-1 alpha and MIP-1 beta preferentially attract CD8+ and CD4+ T cells, respectively, and MIP-1 beta is chemotactic for monocytes, T cells, and NK cells (42, 43). Identification of a robust signature of cytokine induction leading to effective vaccination would be important for LT recipients to prevent the risk of SARS-CoV-2 infection as possible.

This study had some limitations. We focused on the immune status after two-dose vaccination among LT recipients, while excluding antibody levels after the first dose of inactivated SARS-CoV-2 vaccines and dynamic changes data on the immune response. LT recipients who did not vaccinate with SARS-CoV-2 are prone to keep a social distance more. We did not recruit such volunteers for this study. The majority of solid organ transplant recipients had a poor response to the COVID-19 vaccines. The non-responders could reflect the basic immune status of LT recipients to some extent. Robust induction of B-cell and T-cell responses by the third dose of inactivated SARS-CoV-2 vaccine was confirmed in a non-randomized trial among healthcare workers (44). In the future, a longitudinal study with a large cohort is needed to address the sustainability of memory cells stimulated by inactivated vaccines and profile the humoral and cellular responses to the third dose among LT recipients. The small sample size of the healthy controls with or without vaccination limited the robustness of the vaccination evaluation between the different populations, although this was not the main objective of this study. The characteristics and functions of B cell-like T cells and CD3+CD19+ cells were unknown in the development and progression of SARS-CoV-2 vaccination. In further studies, this will be addressed. Despite that, our study demonstrated the varied immune response status among LT recipients after two doses of vaccination, especially in LT recipients with positive neutralizing antibodies (Type 1). More importantly, the signature of antibody response, peripheral immune subsets, plasma cytokine, and chemokine related to effective vaccination were obtained in LT recipients. For LT recipients with negative neutralizing antibodies (Type 2 and Type 3), mild cellular response characteristics were found in some patients, which were crucial for the introduction of an additional (third) dose of the homologous vaccine in the next stage.

## Data availability statement

The original contributions presented in the study are included in the article/**Supplementary Material**. Further inquiries can be directed to the corresponding authors.

## Ethics statement

The studies involving human participants were reviewed and approved by The Ethics Committee of Beijing YouAn Hospital. The patients/participants provided their written informed consent to participate in this study.

## Author contributions

GL, YO, and BD are responsible for the conception and design. BD, GZ, and JZ collected the data and samples. YO and GZ are responsible for the antibody titer and neutralizing antibody detection. YO and WW designed and performed the CyTOF experiments and corresponding analyses. JY detected the cytokine/chemokine/growth factors. YO and BD analyzed and integrated the results and drafted and wrote the manuscript. GL, ML, and DC assisted in modifying the manuscript. All authors are responsible for the review and revision of the manuscript. All authors contributed to the article and approved the submitted version.

## Funding

This work was supported by Beijing Natural Science Foundation (M21006; L202024), Beijing Hospitals Authority Youth Programme (QML20201701), the Key medical professional development plan of Beijing Hospital Authority (ZYLX202124), Reform and Development-Basic and Clinical Cooperation Project (Y-2021YS-2), and the Beijing Municipal Institute of Public Medical Research Development and Reform Pilot Project (JING YI YAN 2021-10).

## Acknowledgments

We would like to thank all subjects who participated in this study.

## Conflict of interest

The authors declare that the research was conducted in the absence of any commercial or financial relationships that could be construed as a potential conflict of interest.

## Publisher’s note

All claims expressed in this article are solely those of the authors and do not necessarily represent those of their affiliated organizations, or those of the publisher, the editors and the reviewers. Any product that may be evaluated in this article, or claim that may be made by its manufacturer, is not guaranteed or endorsed by the publisher.
