# Supplementary material for: Immunogenicity profiling and distinct immune response in liver transplant recipients vaccinated with SARS-CoV-2 inactivated vaccines
Source: Front Immunol. 2022 Sep 14;13:954177. doi: 10.3389/fimmu.2022.954177 (PMC9517166; doi:10.3389/fimmu.2022.954177)
Supplement: Supplementary file 1 [file DataSheet_1.docx]

**Supplementary data**

**Supplementary Table 1 Mass cytometry Panel Design**

| Antigen | Symbol and Mass | Antibody clone | Source |
| --- | --- | --- | --- |
| CD45 | 89 | HI30 | Fluidigm |
| CXCR3 (CD183) | 141 | 49801 | R＆D |
| CD19 | 142 | HIB19 | Fluidigm |
| CCR6 (CD196) | 143 | EPR24590-123 | abcam |
| CD45RO | 144 | UCH-L1 | abcam |
| CD4 | 145 | RM4-5 | Fluidigm |
| CD8 | 146 | RPA-T8 | Fluidigm |
| PSTAT5 | 147 | Y694 | Fluidigm |
| CD16 | 148 | 3G8 | Fluidigm |
| CD34 | 149 | 581 | Fluidigm |
| CD28 | 150 | EPR22076 | abcam |
| IP10 | 151 | [J034D6](https://www.biolegend.com/en-us/search-results?Clone=J034D6) | Biolegend |
| CD11C | 152 | ICRF 3.9 | R＆D |
| PSTAT1 | 153 | 58D6 | Fluidigm |
| CCR7 (CD197) | 154 | 150503 | R＆D |
| PD-1 | 155 | EH12.2H7 | Fluidigm |
| STAT6 | 156 | 253906 | R＆D |
| PSTAT3 | 158 | Y705 | Fluidigm |
| FoxP3 | 159 | PCH101 | Fluidigm |
| CD14 | 160 | 134620 | R＆D |
| CD294 (CRTH2) | 161 | 301108 | R＆D |
| CD69 | 162 | FN50 | Fluidigm |
| CD56 | 163 | NCAM16.2 | Fluidigm |
| CD161 | 164 | HP-3G10 | Fluidigm |
| IFN | 165 | B27 | Fluidigm |
| CD45RA | 166 | HI100 | Fluidigm |
| CD163 | 167 | 215927 | R＆D |
| Ki67 | 168 | Ki67 | Fluidigm |
| CD25 | 169 | 2A3 | Fluidigm |
| CD3 | 170 | SP7 | abcam |
| CD68 | 171 | Y1/82A | Fluidigm |
| Perforin | 172 | B-D48 | abcam |
| HLA-DR | 173 | L243 | Fluidigm |
| CD66b | 174 | 913542 | R＆D |
| CD127 | 175 | EPR2955(2) | abcam |
| Granzyme | 176 | EPR20129-217 | Fluidigm |
| CD11b | 209 | ICRF44 | Fluidigm |

**Supplementary Table 2**. Adverse Events from two dose Inactivated Vaccine (n=46)

| Variables | N（%） | Grade（1-4） |
| --- | --- | --- |
| *Local adverse event* | 5（10.9%） |  |
| Pain | 4 | 1 |
| Erythema | 0 | NA |
| Swelling | 1 | 1 |
| *Systemic adverse event* | 14（30.4%） |  |
| Fever | 1 | 2 |
| Chill | 0 | NA |
| Fatigue | 7 | 0 |
| Myalgia | 5 | 1 |
| Dyspnea | 1 | 2 |
| Arthralgia | 0 | NA |
| Headache | 0 | NA |
| Nausea or vomiting | 0 | NA |
| Diarrhea | 0 | NA |
| Rash | 0 | NA |
